# Interstitial expansion in health and disease - an equilibrium contrast CMR study

**DOI:** 10.1186/1532-429X-14-S1-O23

**Published:** 2012-02-01

**Authors:** Daniel Sado, Andrew Flett, Steven K White, Sanjay M Banypersad, Viviana Maestrini, Atul Mehta, Philip N Hawkins, Derek J Hausenloy, Perry Elliott, James Moon

**Affiliations:** 1 Imaging Centre, The Heart Hospital, London, UK; 2 Inherited Cardiac Disease, The Heart Hosptial, London, UK; 3 Lysosomal Disorderds Unit, The Royal Free Hospital, London, UK; 4 Amyloidosis, National Amyloidosis Centre, London, UK

## Summary

In this study of 278 particiapnts, we aimed to evaluate the cardiac interstitium in health and disease using Equilibrium constrast CMR (EQ-CMR). We found gender and disease differences in the contrast myocardial volume of distribution Vd(m) and correlations with clinical CMR markers of disease.

## Background

Interstitial myocardial volume expansion is an important factor in cardiac disease but until recently could only be accurately assessed with biopsy. We used a new method, EQ-CMR, to accurately quantify the interstitium across a wide spectrum of cardiac diseases.

## Methods

The three steps in EQ-CMR are: 1) a primed gadolinium infusion to achieve contrast equilibrium, 2) Signal (T1) measurement pre and post equilibrium, 3) measurement of blood contrast volume (1- haematocrit). This allows calculation of Vd(m) by:

Vd(m) =(1-hematocrit) x Δ(1/T1)myo ÷ Δ(1/T1)blood.

Vd(m) was measured in 278 subjects: 86 normal subjects (median age 43, range 24 to 81, 51% male) and 192 patients with Anderson-Fabry disease (AFD, n=17), dilated cardiomyopathy (DCM, n=31), hypertrophic cardiomyopathy (HCM, n=31), severe aortic stenosis (AS, n=66), cardiac amyloidosis (n=27) or myocardial infarction (MI, n=20).

## Results

In normal subjects, mean Vd(m) was higher in females (0.274) than males (0.237, P<0.001). In all diseases, Vd(m) was higher than normal subjects (P<0.001) except the intracellular storage disease AFD (0.250, P=0.9). Vd(m) was the same in DCM (0.280), HCM (0.291) and AS (0.276), but higher in the exemplar of infiltrative disease, cardiac amyloidosis (0.466) and higher again in MI (0.585, each P<0.001), (figure). These trends were also present when disease data was compared to gender matched normal subjects. Where Vd(m) was elevated, correlations existed with clinical CMR parameters such as ejection fraction and septal thickness in apparent disease specific patterns, (table).

**Figure 1:**
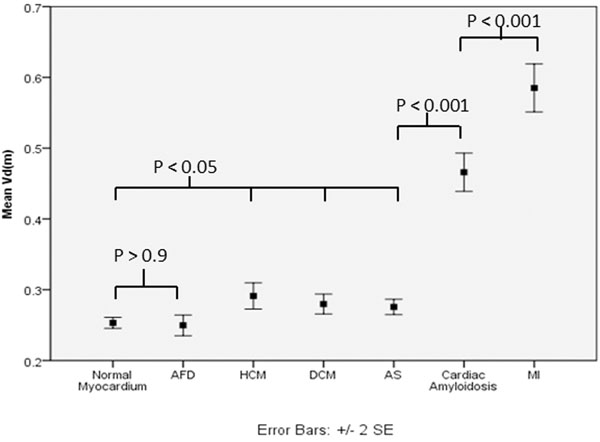


**Table 1:** Pearson Correlations between Vd(m) and CMR markers of disease

Disease	EF	EDV(i)	ESV(i)	Mass(i)	Left Atrial Area (i)	Other
Normal Subjects	NS	NS	NS	R=-0.36**	NS	None
AFD	NS	NS	NS	NS	NS	None
DCM	R=-0.35*	NS	NS	R=-0.36*	R=0.65***	None
AS	NS	NS	R=0.51*	NS	NS	Aortic Valve Area: R=-0.41**
HCM	NS	NS	NS	NS	NS	LGE%: R=0.48*
Cardiac Amyloidosis	R=-0.57**	NS	R=0.63***	R=0.44*	NS	Septal Thickness: R=0.51**

Key: NS: No significant correlation; *p < 0.05; ** p<0.01; *** p<0.001

## Conclusions

This preliminary study suggests that Vd(m) is a potentially important new biomarker across the spectrum of health and cardiac disease.

## Funding

1) The British Heart Foundation (Cardiomyopathy, Myocardial Infarction, Aortic Stenosis and Normal Subjects).

2) Genzyme Coorporation (Anderson Fabry Disease).

3) GlaxoSmithKline (Amyloidosis and Normal Subjects).

